# Model based planning and layout optimization for multilevel express processing centers through A case study of air express sorting center

**DOI:** 10.1038/s41598-025-11113-8

**Published:** 2025-09-30

**Authors:** Mengke Yang, Tao Wang, Jintong Yao, Peigang Qiu, Guangfu Gong

**Affiliations:** 1 Beijing University of Posts and Telecommunications, Bei Jing, 100086 China; 2 Tibet Autonomous Region Postal Administration, Lhasa, 850000 China; 3 State Post Express Logistics Intelligent Equipment Industrial Technology Research Institute, Nanling, 241300 China

**Keywords:** Genetic algorithm, Layout planning, Intelligent optimization, Systematic layout planning, Layout optimization, Layout design, Computer science, Mechanical engineering, Computational science

## Abstract

The construction of multi-level express processing centers constitutes a core strategy for addressing escalating logistical demands, with the layout planning of these hubs significantly impacting the efficiency, cost-effectiveness, and customer satisfaction associated with express delivery services. However, two significant limitations were identified in traditional planning processes: insufficient modeling capabilities and an excessive reliance on experiential knowledge in planning and design. This study proposes a planning model for express parcel handling centers that accounts for multi-level demands. The core of this approach lies in integrating key elements, such as functional area division and correlation values, into the layout planning analysis framework. An improved Genetic Algorithm (GA) is incorporated into the system layout design method, and it realizes automated layout planning of express parcel sorting and handling centers. A case study conducted on an air logistics center demonstrates the feasibility, effectiveness, and applicability of the proposed method. The results indicate that this approach can increase space utilization by 15–28%.This research not only provides innovative perspectives and solutions for layout decision-making in multi-level express processing centers but also offers a framework that can be adapted to complex layout optimization challenges across various industries beyond logistics. By optimizing the layout of express processing centers, it enables faster delivery times, improves resource utilization, and lowers energy consumption, delivering tangible benefits to both businesses and consumers.

## Introduction

Driven by economic globalization and the vigorous development of e-commerce, the express logistics industry is experiencing unprecedented growth^1^. As critical nodes in the express logistics network, multi-level express processing centers play a vital role in package sorting, transfer, and distribution.

However, the increasing volume of business and tightening time requirements pose greater challenges to the layout planning of these centers^2^. Traditional layout planning methods primarily rely on manual experience, making it difficult to adapt to complex and variable operational environments, resulting in low resource utilization and operational inefficiency. Therefore, how to scientifically and reasonably plan the layout of multi-level express processing centers has become a critical issue that needs to be addressed urgently^3^. This leads to the following research questions:

RQ1: How can a comprehensive planning model be developed to optimize the layout of express delivery processing centers at different levels?

RQ2: What key factors should be quantified in the planning process?

RQ3: Which algorithm can be adopted to ensure the feasibility, effectiveness, and applicability of the planning?

In response to this issue, researchers have conducted extensive and in-depth exploration in the field of logistics center layout planning. The Systematic Layout Planning method has been widely applied in facility layout design due to its systematic and practical nature^4–6^. Additionally, multi-objective planning models have been introduced to optimize logistics center layouts, balancing multiple objectives such as cost and efficiency^7^. For different types of logistics centers, such as railway container logistics centers^8^ industrial gas packaging logistics centers^9^ express processing centers^10^ and hazardous material logistics ports^11^ researchers have also proposed targeted layout planning methods. Meanwhile, some scholars have attempted to apply advanced optimization algorithms to logistics center layout optimization, such as the improved dung beetle algorithm^12^. In terms of logistics park planning, existing research has also focused on the impact of factors such as traffic and community, and proposed optimization strategies such as two-stage layout methods^13^ and improved genetic algorithms^14,15^.

To effectively address these issues, this study innovatively proposes a planning layout method based on an integrated planning model of multi-level express processing centers. This method incorporates key elements such as functional area size and relationship values into the layout planning analysis system, ensuring the scientific and practical nature of the planning. This concept aligns with the idea proposed by Wang et al.^16^ in logistics park planning, where they comprehensively considered factors such as traffic and community to reduce logistics costs and environmental pollution. To further enhance the applicability of the model, this study references a large number of cutting-edge research. Marian and Putz^17^ on generative design in factory layout planning and Perrone et al.^18^ on emerging technologies in dynamic layout planning introduce innovative technological concepts. Zhang et al.^19^ studied the functional area layout of railway logistics parks. The case of Su et al.^20^ combining the SLP method and genetic algorithm to optimize cabin layout provides a reference for this study to construct a mathematical planning model and corresponding solution algorithm using a combination of systematic layout planning methods and genetic algorithms. Chen Dengkai et al. introduced a fuzzy evaluation method into the genetic algorithm to analyze the functional adjacency between compartments and personnel flow relationships, thereby optimizing the layout of submarine living quarters^21^. Fan, ZS utilized a genetic algorithm (GA) to automatically generate MHRB layouts, reducing the heavy reliance on designers’ experience during the design process, which addresses concerns regarding design efficiency and quality^22^. Pasumarthi proposes a method for optimizing the assembly zone of modular robots in heterogeneous obstacle environments. The method uses a multi-objective genetic algorithm to minimize total travel distance and individual distance disparities^23^.

Sun Jianwei’s^24^ analysis of logistics center layout planning emphasizes the critical role of logistics center in the supply chain. Wang’s^16^ research on intelligent logistics park planning layout based on an improved genetic algorithm provides practical guidance for application. The edge collaboration technology for optimizing railway logistics center layout proposed by Zhang and Ahmed^25^ broadens the technical perspective of logistics center layout optimization. Zhen et al.‘s^26^ research on dynamic path planning for third-party logistics centers provides a new perspective on logistics center layout from a unique path planning angle. Additionally, Takata et al.‘s^14^ research on solar energy utilization in logistics centers provides beneficial insights for the development of green logistics. Ömer Faruk Yılmaz provides mathematical models and optimization methods for logistics layout planning, assisting decision-makers in determining the optimal warehouse layout schemes to enhance the overall efficiency of supply chain operations^27^.

In summary, despite significant progress in the field of logistics center layout planning, existing research presents limitations concerning planning models. First, much work centers on single-scenario optimization, neglecting the holistic layout optimization of multi-level parcel hub networks. Second, current planning models seldom comprehensively integrate the multifaceted influencing factors. Unlike existing research, this study focuses on the layout planning problem specific to multi-level express processing centers, and by constructing an integrated planning model for these hubs, aims to derive the optimal layout configuration through optimization algorithms.

This study has made several significant advancements in the field of express delivery layout planning. Theoretically, by introducing a comprehensive planning model that integrates functional area allocation, correlation values, and the Systematic Layout Planning (SLP) framework, it successfully overcomes the limitations of existing models that focus solely on single-scenario optimization while neglecting multidimensional influencing factors. Methodologically, the study significantly enhances the efficiency and effectiveness of layout optimization by employing an improved Genetic Algorithm to solve the proposed planning model. Experimental results clearly demonstrate the superior performance of the method in achieving optimal layout configurations; the organic integration of Systematic Layout Planning with the improved Genetic Algorithm effectively addresses complex layout challenges. Furthermore, through a case study of an air logistics center, this research provides practical guidance for the layout design of multi-level express parcel handling centers. The practical validation results show that, compared to traditional manual layout methods, the proposed optimization approach can significantly improve space utilization by 15–28%, delivering substantial economic benefits for related enterprises in terms of operational efficiency, resource utilization, and cost savings.

### **Facility layout planning using systematic layout planning**

#### Research methodology and approach

The central challenge in hub-level parcel hub layout planning research lies in constructing an optimized solution planning model and intelligent algorithm, grounded in layout planning theory, using the Systematic Layout Planning methodology. This entails addressing key issues such as functional area demarcation, area estimation for each functional area, and the determination of relational values between zones. To tackle this problem, the study employs a combined SLP framework and material flow analysis, delineating functional areas based on the SLP method and determining inter-zonal logistical relationships via material flow analysis. Simultaneously, it adopts an efficiency-driven approach to area estimation, estimating the required area for each functional zone based on target processing efficiency and logistics equipment selection. The research approach integrates comprehensive consideration of inter-functional area relational values, area estimation outcomes, and factors such as equipment selection to construct an optimized solution planning model and develop an intelligent algorithm. This provides both theoretical support and a practical foundation for the layout design of hub-level express processing centers.

#### Hub-level express parcelProcessing center layout requirements analysis

To effectively address the planning and layout challenges of hub-level express processing centers, it is essential to clearly define their spatial requirements. Typically, the interior space of such centers is divided into functional areas, each supporting a specific stage of the parcel sorting process. A retrospective analysis of the developmental history and contemporary structure of these centers reveals that Road Freight Terminals are not only prevalent but also dominant in the sector. Consequently, this study focuses on the fundamental operational processes characteristic of road-type hub-level express processing centers.

Upon arrival at the sorting center, parcels are initially unloaded and subjected to inbound scanning, which encompasses information registration, security inspection, and disinfection protocols. Subsequently, parcels undergo primary sorting based on their type, followed by further segregation in the automated small parcel sorting area or the automated matrix sorting area for large parcels, according to size and destination, with subsequent parcel consolidation and repackaging. Simultaneously, non-standard and specialized parcels are processed manually in the special parcel handling area. Finally, the sorted parcels are subjected to outbound scanning and repackaging before being loaded onto transport vehicles for dispatch.

Despite functional variations, hub-level express processing centers commonly exhibit the following attributes: they function as critical logistics nodes, rely on information systems, possess inherent sorting capabilities, and demonstrate similar spatial planning principles.

#### Forecast of express handling capacity

**Fig. 1:**
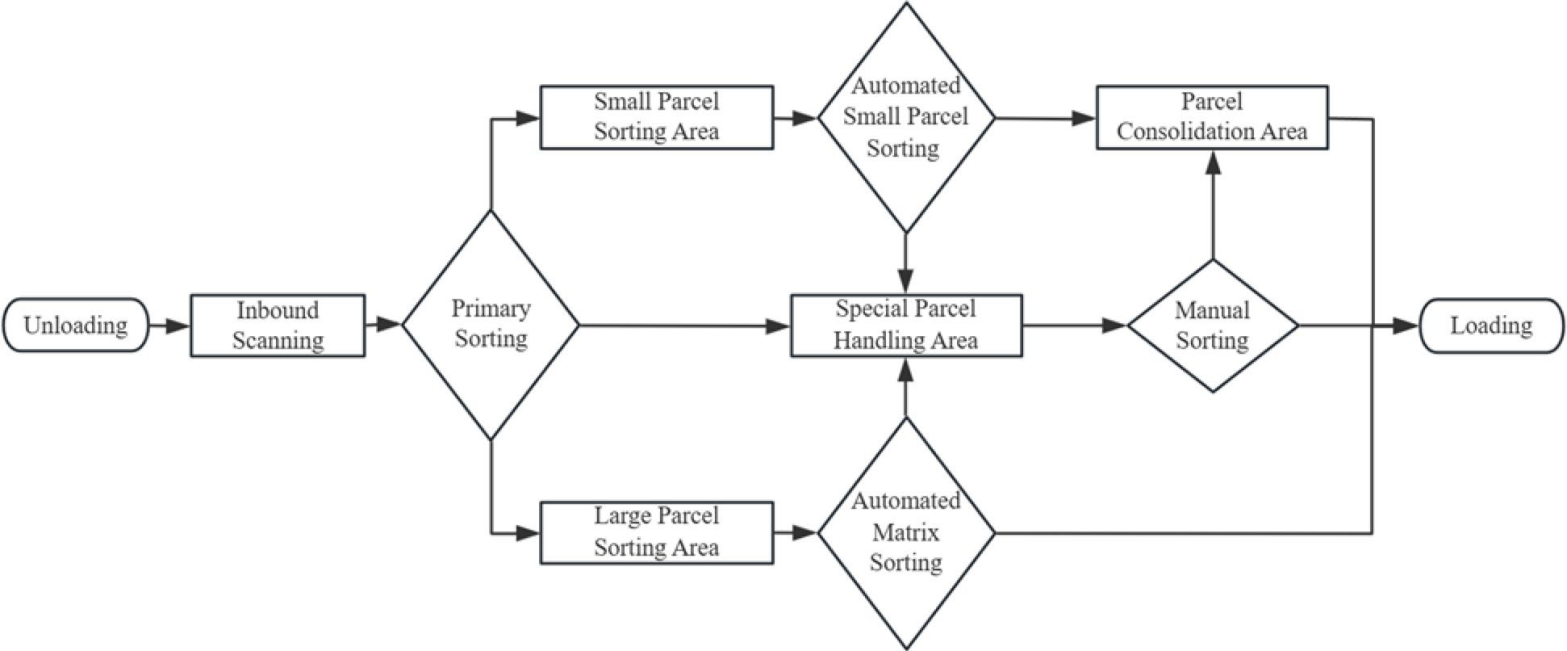
Express Parcel Sorting Flowchart.

The design processing capacity is usually based on the forecast of express demand in the next five years, which should align with the facility’s lifespan. Given the relatively stable pattern of express demand, historical data can be utilized for future demand prediction. Since global express volumes show an upward trend, using a trend-adjusted exponential smoothing method to predict future demand is appropriate. It is recommended to employ a combination forecasting method that incorporates local overall express throughput data and market share to determine the design processing capacity. The package sorting flowchart is shown in Fig. 1.

It is also worth noting that forecasting accuracy is typically higher when using aggregate rather than disaggregate forecasting methods. Therefore, when selecting historical data, it is advisable to use the total local parcel processing volume rather than data from a specific express delivery company. Once the forecast is completed, the required design processing capacity of the facility can be calculated based on the projected market share.

#### Selection of logistics equipment and calculation of function area size

A parcel sorting center, primarily tasked with sorting operations, serves as a crucial logistics hub. Its fundamental operational stages encompass unloading, sorting, security inspection, unpacking, building parcels, loading, storage, recycling, and information processing, each of which is typically executed in dedicated functional areas. The determination of area size for each operational zone necessitates calculations based on several factors, including the area’s function, the operational methodology, the chosen equipment, and the intensity of logistics.

In consideration of equipment and comparable center equipment selection plans approved by the company, it is imperative to choose suitable logistics processing equipment. Typically, each operation is conducted within a distinct functional area. The size of the operational area, however, should be determined based on several factors, such as the area’s function, the operational mode, the equipment selection, and the intensity of logistics.

The functional area layout of a parcel hub is a critical component of its operational efficiency, with each area designed to support specific tasks and processes. The loading and unloading area serves as the primary input and output point in a parcel hub’s logistics, with area $\:{S}_{1}$ dependent on the number of platforms, logistics intensity, and equipment selection. The express temporary storage area is a crucial buffer addressing peak periods, delays, and damaged items, with area $\:{S}_{2}$​ correlating with storage capacity and equipment footprint. The primary sorting area (or coarse sorting area) is used for initial scanning and sorting, and area $\:{S}_{3}$​ is determined by factors like the volume of express items processed per operation, per unit area, and processing efficiency per unit time $\:{p}_{c1}$​.The large parcel sorting area and artificial sorting area handle oversized and irregularly shaped items, respectively, with area estimation methods for $\:{S}_{4}$ and $\:{S}_{5}$ analogous to those used for the primary picking area.The small parcel sorting area processes small to medium-sized parcels, and area $\:{S}_{6}$ encompasses sorting, packaging, logistics information entry, and high-speed sorting zones.The comprehensive office area, including employee offices, rest areas, and equipment control, is estimated as $\:{S}_{7}$​. Express processing centers in aviation and high-speed rail hubs necessitate a dedicated security zone, with an area of $\:{S}_{8}$.A packaging and processing area, for logistics packaging compliant with transportation requirements or value-added service processing, is required in certain hub-level facilities, with an estimated area of $\:{S}_{9}$.The warehousing sector facilitates goods storage and value-added services, with the area $\:{S}_{10}$​ dependent on storage capacity and equipment occupancy. The area for other functional areas $\:{\:S}_{11}$ in hub-level express processing centers can be calculated using the number of equipment (or personnel) multiplied by the unit area required. Hub-level express processing centers have various other types of functional areas according to their type and services provided. It is not possible to list all the functional areas here, but the area calculation of these functional areas can use a general method, that is, the number of equipment (or personnel) multiplied by the unit area required per equipment (or person). Estimated area for other functional areas $\:{\:S}_{11}$.

The precise formula for the calculation is provided in Table 1.

**Table 1:** Functional area calculation table.

Basic Functional Areas	Formula
Loading and Unloading Area	$\:{S}_{1}=\frac{{Q}_{1}\text{*}{s}_{a}}{{T}_{1}\text{*}{p}_{a}}\:$ (1)
Express Temporary Storage Area	$\:{S}_{2}=\frac{{Q}_{2}\text{*}{v}_{b}}{{p}_{b}}+{S}_{b}$ (2)
Primary Sorting Area	$\:{p}_{c1}=\frac{{Q}_{3}}{{T}_{3}}$ (3)
	$$\:{S}_{3}=\frac{{Q}_{3}}{{p}_{c2}}\:\:\:\:\:\:\:\:\:\:\:\:\:\:\:\:\:\:\:\:\:\:\:\:\:\:\:\:\:\:\:\:\:\:\:\:\:\:\:\:$$ (4)
Small Sorting Area	$\:{S}_{6}={S}_{d1}+{S}_{d2}\:\:\:\:\:\:\:\:\:\:\:\:\:\:\:\:\:\:\:\:\:\:\:\:\:\:\:\:\:\:$ (5)
	$$\:{Q}_{e}={p}_{e1}-{p}_{e2}\:\:\:\:\:\:\:\:\:\:\:\:\:\:\:\:\:\:\:\:\:\:\:\:\:\:\:\:\:\:\:\:\:\:\:$$ (6)
Comprehensive Office Area	$\:{S}_{7}={U}_{f1}\text{*}{s}_{f1}+{U}_{f2}\text{*}{s}_{f2}$ (7)
Security Inspection Area	$\:{S}_{8}={U}_{g}\text{*}{s}_{g}+{S}_{g}$ (8)
Packaging and Processing Area	$\:{S}_{9}={U}_{h}\text{*}{s}_{h}$ (9)
Storage Area	$\:{S}_{10}=\frac{{Q}_{10}\text{*}{v}_{i}}{{p}_{i}}+{S}_{i}$ (10)
Other Functional Areas	$\:{S}_{11}={U}_{j}\text{*}{s}_{j}+{S}_{i}$ (11)
$\:{Q}_{1}$represents the logistics volume of a single loading and unloading operation; $\:{s}_{a}$is the required area for a single loading and unloading platform; $\:{T}_{1}$is the specified time for a single loading and unloading operation; $\:{p}_{a}$is the loading and unloading efficiency of a single loading and unloading platform; $\:{Q}_{2}$represents the average storage volume of the temporary storage area per unit time; $\:{v}_{b}$denotes the space occupied by a unit storage volume; $\:{p}_{b}$is the storage space efficiency per unit area; $\:{S}_{b}$refers to the area required for other functional devices; $\:{p}_{c1}$represents processing efficiency per unit time; $\:{\:Q}_{3}$represents the logistics volume of the stage sorting operation; $\:{T}_{3}$represents the specified time for the stage sorting operation; $\:{p}_{c2}$represents the efficiency of parcel processing per unit area under the device selection scheme for achieving$\:{p}_{c1}$; $\:{S}_{d1}$denotes the area of the sorting operation zone, calculated in a manner identical to that of the primary sorting zone; $\:{S}_{d2}$signifies the area of the unpacking zone, computed using the same method as for the parcel temporary storage zone; $\:{p}_{e1}$represents the efficiency of logistics input; $\:{p}_{e2}$represents the efficiency of sorting and supply; $\:{U}_{f1}$represents the average number of office personnel per unit time; $\:{S}_{f1}$is the per capita office space, typically taken as $4.5\sim 7m^{2}$; $\:{U}_{f2}$denotes the number of information system devices; $\:{S}_{f2}$is the area occupied by each device, generally set at $4.5\sim 5.5m^{2}$; $\:{U}_{g}$represents the number of security checkpoints; $\:{s}_{g}$is the area required per unit security checkpoint; $\:{S}_{g}$is the area of the emergency isolation zone; $\:{Q}_{10}$is the design storage capacity of the storage area; $\:{v}_{i}$is the space occupied by a unit of storage; $\:{p}_{i}$is the storage space efficiency per unit area; $\:{S}_{i}\:$is the area required for other functional equipment; $\:{U}_{j}$is the number of equipment (or personnel) required for the functional area; $\:{s}_{j}$is the area required per unit of equipment (or personnel); $\:{\:s}_{i}$is the area required for other functional equipment.	

### Multi-level express processing center layout planning model

#### SLP method application

**Fig. 2:**
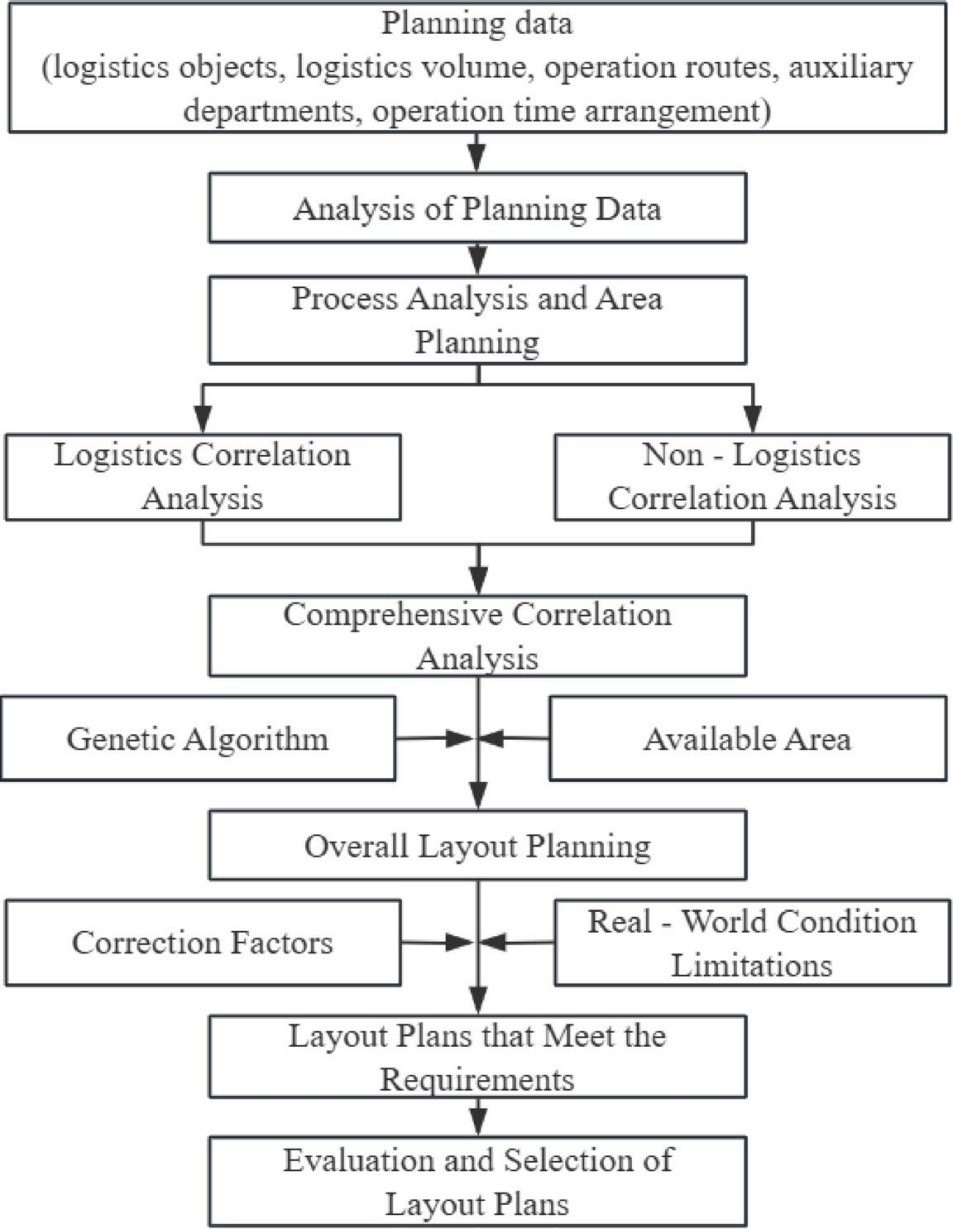
SLP Method Flowchart.

The main application process of the Systematic Layout Planning (SLP) method is shown in Fig. 2. When using the SLP method, it is first necessary to collect planning data for the corresponding facility, such as the logistics objects, material flow, logistics operation routes, auxiliary departments, and work schedules. This data is then used to carry out preliminary process analysis and area planning.

Based on the data obtained previously, a correlation analysis between areas is conducted, which includes both logistics correlation analysis and non-logistics correlation analysis. Logistics correlation analysis is performed by analyzing the material flow and logistics routes of facility operations to determine a logistics intensity value between each area pair. This value is used to measure the strength of the logistical relationship between those two areas. This is typically represented using a From-To Chart.

Non-logistics correlation analysis is conducted by analyzing the strength of other business connections between areas arising from non-logistical activities within the operational processes. Based on actual needs, corresponding standards are established to evaluate the strength of non-logistical relationships between areas. After obtaining the logistical and non-logistical relationships between areas, they need to be combined according to their respective weights to derive a quantitative value for the overall correlation between each area pair.

#### The mathematical model

Based on the actual survey, the following assumptions are made for establishing the layout optimization model of the express parcel sorting center:

(1) The express parcel processing center and each functional zone are assumed to be rectangular block structures. The length and width of the processing center are known, while each functional zone has a fixed minimum area with adjustable length and width.

(2) The layout plane for the functional zones is assumed to be coplanar.

(3) The inbound and outbound points are assumed to be located at the midpoint of each piece of equipment.

(4) The model’s parameters, decision variables, reference lines, and coordinate system are established as illustrated in the figure.

In this stage of the planning model, the building area to be planned can be considered a rectangle with a length of L and a width of W. There are n functional areas to be arranged in the parcel hub, and all functional areas are rectangular. If i represents the functional area number, then $\:{x}_{i}$ represents the horizontal coordinate of the geometric center of the functional area, $\:{y}_{i}\:$represents the vertical coordinate of the geometric center of the functional area, $\:{l}_{i}$ represents the length of the functional area, and $\:{w}_{i}$ represents the width of the functional area.

**Fig. 3:**
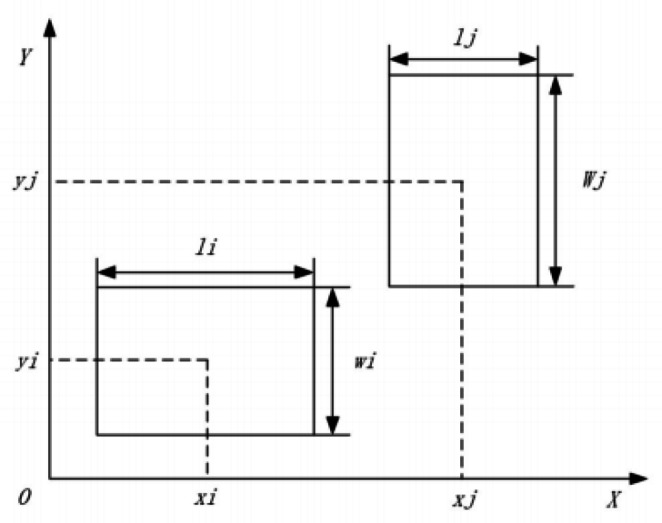
Planar Schematic Diagram of The Model.

#### Objective function

The objective of hub-level express processing center layout planning, utilizing the SLP method, is to minimize the total logistics cost ($\:{F}_{1}$) while simultaneously maximizing the overall comprehensive relationship ($\:{F}_{2}$).

12 $$\:\text{min}\:{F}_{1}=\mathop{\sum\:}\limits_{i=1}^{n}\mathop{\sum\:}\limits_{j=1}^{n}{c}_{ij}{f}_{ij}{d}_{ij},$$

13 $$\:\text{max}\:{F}_{2}=\mathop{\sum\;}\limits_{i=1}^{n}\mathop{\sum\:}\limits_{j=1}^{n}{T}_{ij}{b}_{ij}\:.\:\:\:\:\:$$

In the formula, $\:n$ is the number of functional areas; $\:i$ and $\:\:j\:$are the numbers of functional areas ($\:i<j$);$\:{c}_{ij}$ represents the unit transportation cost; $\:{f}_{ij}$ is the logistics volume;$\:{d}_{ij}$ represents the Manhattan distance.$\:{d}_{ij}=|{x}_{i}-{x}_{j}|+|{y}_{i}-{y}_{j}|$;$\:{T}_{ij}$ is the comprehensive correlation of the functional areas; $\:{b}_{ij}$is the adjacency coefficient between functional areas, obtained by converting $\:{d}_{ij}$ (Table 2).

**Table 2:** Adjacency coefficient Table.

Functional Area Adjacency Coefficient	Distance Interval $\:{\varvec{d}}_{\varvec{i}\varvec{j}}$
1.0	$\:\left[0\right.,\left.\:\frac{{d}_{max}}{6}\right)$
0.8	$\:\left[\frac{{d}_{max}}{6}\right.,\left.\:\frac{{d}_{max}}{3}\right)$
0.6	$\:\left[\frac{{d}_{max}}{3}\right.,\left.\:\frac{{d}_{max}}{2}\right)$
0.4	$\:\left[\frac{{d}_{max}}{2}\right.,\left.\:\frac{{2d}_{max}}{3}\right)$
0.2	$\:\left[\frac{{2d}_{max}}{3}\right.,\left.\:\frac{5{d}_{max}}{6}\right)$
0	$\:\left[\frac{5{d}_{max}}{6}\right.,\left.\:{d}_{max}\right)$

In order to be used for intelligent algorithm solutions, it is necessary to convert multi-objective functions into single-objective functions. We normalize the objective functions $\:{F}_{1}$and $\:{F}_{2}$ with different dimensions, and the final single objective function $\:F$ is:.

14 $$\:\text{min}\:F=\frac{{\sum\:}_{i=1}^{n}{\sum\:}_{j=1}^{n}{c}_{ij}{f}_{ij}{d}_{ij}}{{\sum\:}_{i=1}^{n}{\sum\:}_{j=1}^{n}{c}_{ij}{f}_{ij}{d}_{max}}-\frac{{\sum\:}_{i=1}^{n}{\sum\:}_{j=1}^{n}{T}_{ij}{b}_{ij}}{{\sum\:}_{i=1}^{n}{\sum\:}_{j=1}^{n}{T}_{ij}}\:,\:\:\:\:\:\:$$

In the formula, $\:{d}_{max}$ represents the sum of the lengths of the longer and shorter sides in the planning area of the express sorting center.

#### Constraints

To ensure the mathematical model aligns with practical planning and precludes unfeasible layout schemes, it must satisfy the following conditions:

Functional areas must not overlap. There must be no overlapping parts between functional areas:

15 $$\:\left|{x}_{i}-{x}_{j}\right|\ge\:\frac{{l}_{i}+{l}_{j}}{2}\:,\:\:\:\:\:\:$$

16 $$\:|{y}_{i}-{y}_{j}|\ge\:\frac{{w}_{i}+{w}_{j}}{2}\:\:.\:\:\:\:\:$$

Functional areas must not exceed the planning scope. The scope of each functional area cannot extend beyond the planning scope of the express sorting center:

17 $$\:\frac{{l}_{i}}{2}\le\:{x}_{i}\le\:L-\frac{{l}_{i}}{2}\:,\:\:\:\:\:\:$$

18 $$\:\frac{{w}_{i}}{2}\le\:{y}_{i}\le\:W-\frac{{w}_{i}}{2}\:\:.\:\:\:\:\:$$

The area of functional areas must meet the conditions. The area of each functional area must at least meet the pre-estimated area of the functional area:

19 $$\:{l}_{i}\text{*}{w}_{i}\ge\:{s}_{i}\:\:\:.\:\:\:\:\:$$

The receiving and dispatching areas are located at the edge. The receiving and dispatching areas need to conduct logistics activities with external vehicles, so they need to be placed at the edge of the facility:

20 $$\:{x}_{k}=\frac{{l}_{k}}{2}\:\:or\:\:{x}_{k}=L-\frac{{l}_{k}}{2}\:or\:\:{y}_{k}=\frac{{w}_{k}}{2}\:or\:\:{y}_{k}=W-\frac{{w}_{k}}{2}$$

Functional area aspect ratio constraints. To avoid unreasonably long and narrow or short and wide shapes of the functional areas, it is necessary to set constraints on the aspect ratio of the functional areas:

21 $$\:\frac{1}{\gamma\:}\le\:\frac{{l}_{i}}{{w}_{i}}\:\le\:\gamma\:\:,(\gamma\:\ge\:1)\:$$

In the formula, γ represents the limiting proportionality coefficient.

#### Solving with genetic algorithm

**Fig. 4:**
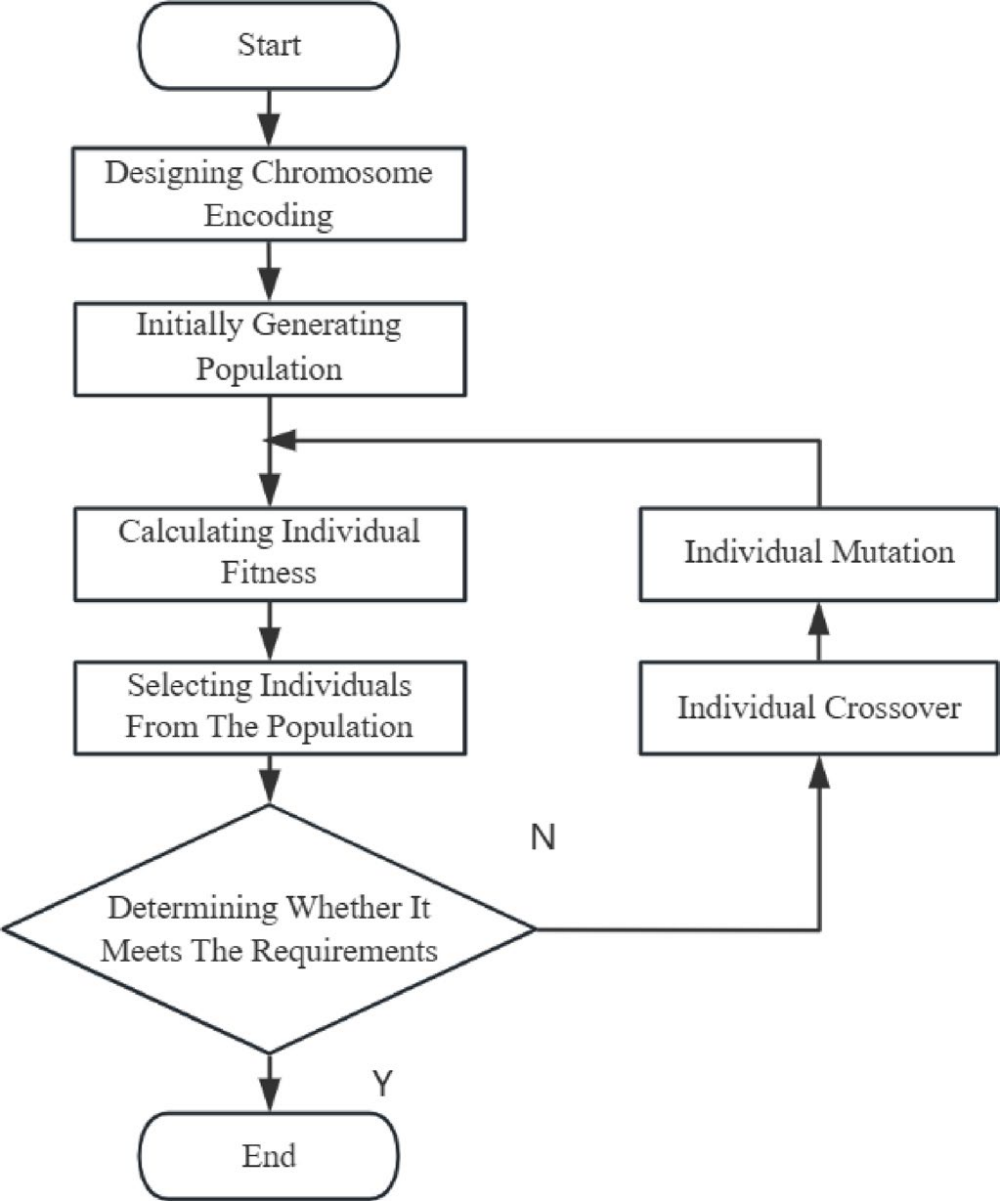
Flowchart of the Genetic Algorithm.

The selection of genetic algorithms (GAs) for the multi-level express distribution center layout problem investigated in this paper is motivated by the following considerations. Multi-level express distribution center layout planning presents a complex multi-objective optimization problem, involving decisions related to the size of multiple functional areas and their inter-relationships, resulting in a vast and intricate solution space. GAs, by mimicking the process of biological evolution through genetic operations such as crossover and mutation, can effectively achieve a global search of the solution space and avoid becoming trapped in local optima, making them particularly well-suited for addressing such complex optimization challenges. Compared to traditional layout optimization algorithms, GAs exhibit greater adaptability and robustness in handling layout planning problems involving multiple levels and constraints. More importantly, in express distribution center layout planning, discrepancies often exist between the objective function and reality. GAs do not impose strict requirements on the form of the objective function; therefore, they can provide effective solutions even when the objective function is complex or includes factors that are difficult to quantify. For these reasons, this paper employs GAs as an effective approach for solving the multi-level express distribution center layout planning problem. The flowchart of the algorithm used is shown in Fig. 4.

#### Chromosome Encoding

In the algorithm of this model, chromosome encoding is facilitated through the use of real number encoding. Specifically, the attributes of an individual functional area are depicted as a gene, and the comprehensive layout plan is characterized as a chromosome. The gene format in this research algorithm is delineated as follows:

22 $$\:{(x}_{i}{,y}_{i},{\lambda\:}_{i})\:\:\:\:\:\:\:\:\:\:\:\:\:\:\:\:\:\:\:\:\:\:\:\:\:\:\:\:\:\:\:\:\:\:\:\:\:\:\:\:\:\:\:\:\:\:\:\:\:\:\:\:\:\:\:\:\:\:\:\:\:\:\:\:\:\:\:\:\:\:\:\:\:\:\:\:\:\:\:\:\:\:\:\:\:\:\:\:\:\:$$

where _i_ is the horizontal coordinate of the functional area’s center point; _i_ is the vertical coordinate of the functional area’s center point; and _i_ is the aspect ratio of the functional area.

23 $$\:{\lambda\:}_{i}=\frac{{w}_{i}}{{l}_{i}}$$

#### Initial Population Setup

In this model, the initial population is generated using a random generation method. Constraint conditions are set within the random generation range to ensure the initial population is closer to feasible solutions; for example, the center points of functional areas are generated within the planning area.

24 $$\:\text{f}\text{i}\text{t}\text{n}\text{e}\text{s}\text{s}\:=\frac{1}{F+m*P}$$

where *m* is the number of violated constraint conditions; and *P* is the penalty coefficient.

- Fitness Function Calculation

In the second stage of this model, the objective function seeks to minimize a positive value. Therefore, the reciprocal transformation method is used to derive the fitness function. Constraint terms in the model are addressed by combining a penalty function method with the fitness function.

- Selection

In this model, tournament selection is used to choose individuals to retain for the next generation.

- Crossover and Mutation.

In this model, a multi-point crossover method is used to form new individuals through gene mating and recombination.

- Termination Condition

The termination condition for the algorithm in this model is reaching a predetermined number of iterations. The algorithm then outputs the best individual, the fitness value of the best individual, and the iteration number in which the best individual first appeared.

## A case study on the planning and layout of an air express sorting center

### Case analysis

A certain logistics and express delivery company plans to establish a new aviation hub-level express processing center in a new airport within a transportation hub city in the southern region of China. This center will be used for receiving and dispatching domestic and international air express within the service area of the aviation hub.

The domestic and international air express sorting, processing, and dispatching facilities of the aviation hub-level express processing center are located in the northern cargo area of the airport, with a length of 700 m, a width of 330 m, and a height of 21 m. The domestic and international air express sorting, processing, and dispatching facilities are directly connected to the southern airport freighter apron positions and to the airport cargo expressway on the north side, ensuring smooth loading, unloading, and transportation operations of express packages entering and leaving the sorting, processing, and dispatching facilities.

### Demand forecast

The demand level smoothing index α is 0.1, and the demand trend smoothing index β is 0.2. Based on the domestic and international air express logistics volume data over the past six years the express demand of the air hub-level express processing center is predicted, and a forecast value table (Table 3) is generated.

**Table 3:** Predicted demand for express Parcels.

	Regional Annual Express Logistics Volume Prediction (Ten Thousand Tons)	Annual Express Demand Forecast (Ten Thousand Tons) at the Express Processing Center
2024	285	143
2025	294	147
2026	302	151
2027	311	156
2028	319	160

### Equipment selection

In aviation hub-level express processing centers, the paramount consideration is ensuring the timely processing of services, given the high added value of air express packages. Consequently, these centers typically opt for mature and reliable, highly automated sorting systems during the selection of their sorting equipment. For instance, matrix sorting systems are employed for preliminary sorting, using tilt-tray sorters or sliding shoe sorters for fully or semi-automated operations respectively. In secondary sorting areas, double-layer cross-belt sorters are deployed—one layer designated for large packages and the other for smaller ones—to facilitate efficient and precise package sorting. To maintain compliance with International Air Transport Association (IATA) and national aviation safety standards, vital inspection equipment such as X-ray security inspection machines and explosive detection apparatus are utilized. Additionally, loading and unloading equipment, including platforms and elevators, are implemented to expedite the loading and unloading of goods.

### Process analysis and functional area division

The processing workflow for inbound express parcels is as follows:

(1) Unloading Zone: Initially, express parcels are unloaded in the designated unloading area.

(2) Security Inspection Area: Following unloading, all parcels undergo a rigorous security inspection process, encompassing scanning, recording, and security validation. Parcels failing to meet security assessment criteria are transferred to a detention area for further customs inspection. Parcels that successfully pass the security inspection Area proceed to the primary sorting area.

(3) Primary Sorting Area: Within the primary sorting area, parcels are processed according to their status: Intercepted, damaged, or otherwise anomalous parcels are routed to a processing center for specialized handling. Oversized or irregularly shaped parcels are directed to a manual sorting area. Remaining standard parcels that have successfully passed security are allocated to the secondary sorting area based on their dimensions and weight.

(4) Secondary Sorting Area: In the secondary sorting area, parcels are categorized according to their destination flight. Any anomalies detected during this process are redirected to the processing center for further examination.

(5) Processing Center: The processing center is responsible for handling anomalous parcels originating from both the primary and secondary sorting areas. Once processing is complete, parcels ready for outbound transport are returned to the manual sorting area for re-sorting based on flight assignments.

(6) Manual Sorting Area: The manual sorting area receives oversized or irregularly shaped parcels from the primary sorting area, as well as parcels returned from the processing center, for sorting according to flight assignments.

(7) Merging and Packaging Area: Subsequently, all sorted parcels converge at the merging area for Radio-Frequency Identification tagging. Parcels requiring consolidation undergo packaging utilizing techniques specifically tailored for air transportation.

(8) Temporary Storage: Upon completion of packaging, parcels requiring temporary storage are transported via forklift to the designated temporary storage facilities.

(9) Loading Area: Ultimately, parcels are transported to the loading area via tractor, prepared for transport to the next destination.

**Fig. 5:**
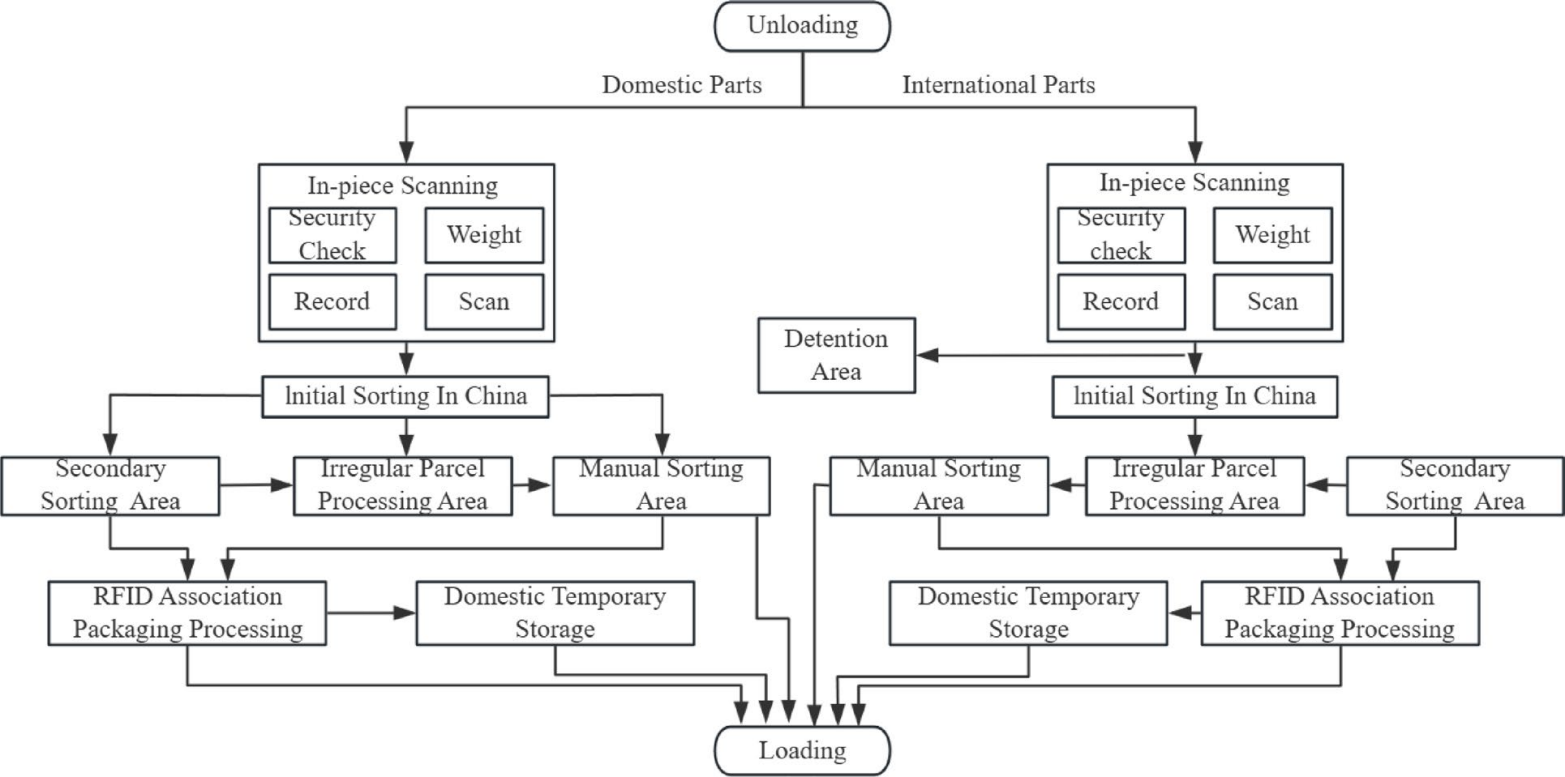
Air Logistics Flow Diagram.

Upon examining the operational procedures of the aviation hub’s express processing center and factoring in its logistical demand scale, we can construct an internal operational flowchart for the express processing center. This is depicted in Fig. 5.

The express processing center at the aviation hub is tasked with offering a myriad of services, encompassing unloading and loading, sorting, security checks, manual sorting, packaging processing, temporary storage, detention, and comprehensive services, among others. These services are divided into a total of 14 functional areas. To address the layout planning problem, a multi-level planning layout method will be employed to analyze the layout planning elements of these functional areas.

### Process analysis and functional area division

**Fig. 6:**
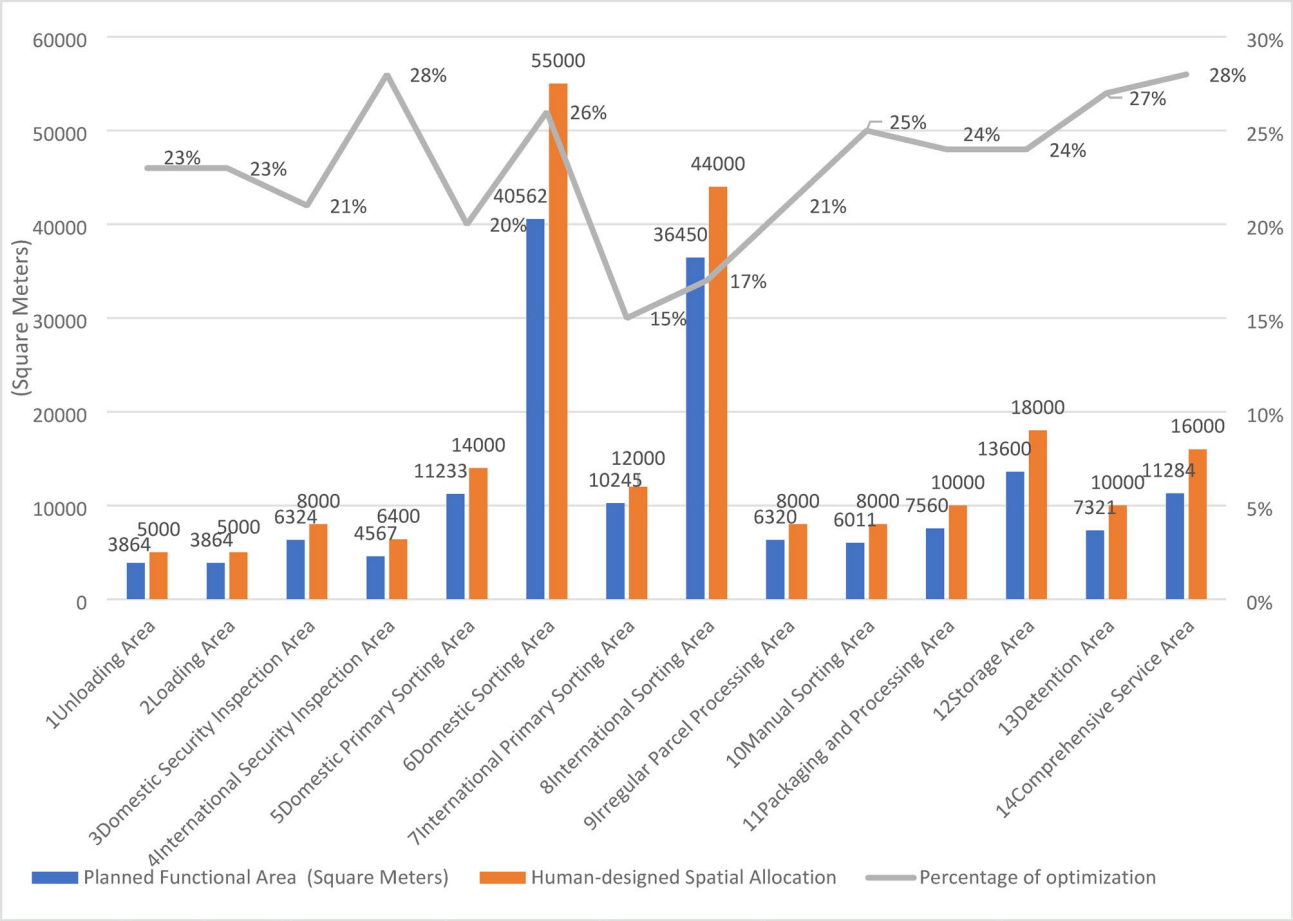
Planned Functional Area.

Based on the demand forecast and equipment selection, the area for each functional area is calculated and determined to be 220,000 m. The data are subsequently summarized in Fig. 6.

According to this calculation method based on equipment efficiency per unit area, the required total area is reduced by 23% compared with manual experience estimation.

### Comprehensive relationship analysis of functional areas

Since logistics factors have a significant impact on the layout planning of aviation hub-level express processing centers, the ratio of the relative importance weights of logistics relationships to non-logistics relationships is taken as 2:1. The comprehensive relationship closeness is calculated based on the formula and the comprehensive relationship diagram between functional areas is obtained, as shown in Fig. 6.

**Fig. 7:**
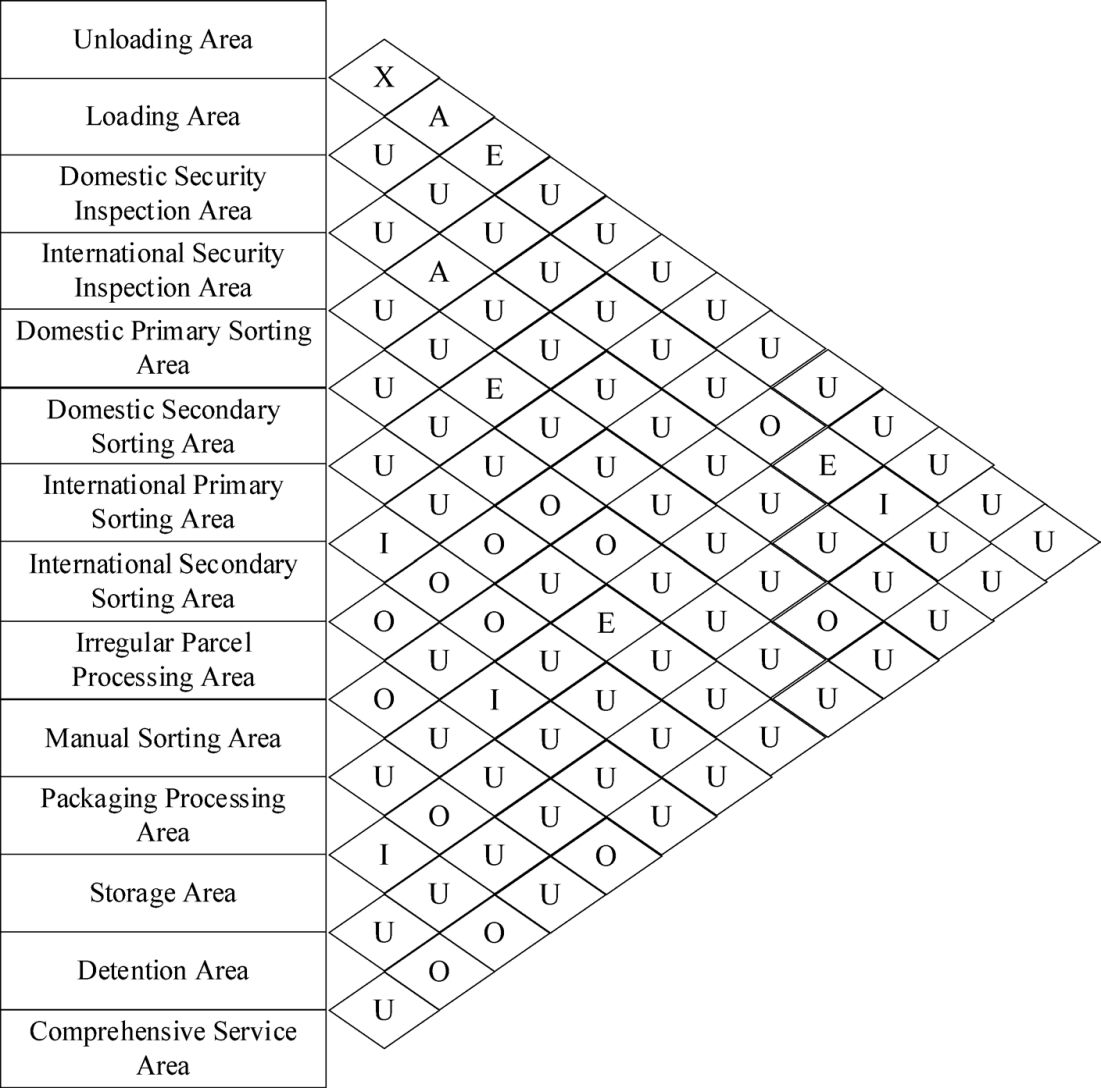
Comprehensive Relationship Diagram of Functional Intervals.

## Results

The genetic algorithm parameters, as set for this study, are shown in Table 4.

**Table 4:** Table of genetic algorithm parameters.

Parameters	parameter values
iteration times	10,000
population size	20
crossover probability	0.2
mutation probability	0.1
penalty coefficient	10,000

By inputting the correlation values between functional areas, area requirements, and boundary constraints, the genetic algorithm generates the center coordinates and aspect ratios of each functional area. The planning region is then scaled to actual dimensions through a normalized coordinate system, as shown in Fig. 7.

**Fig. 8:**
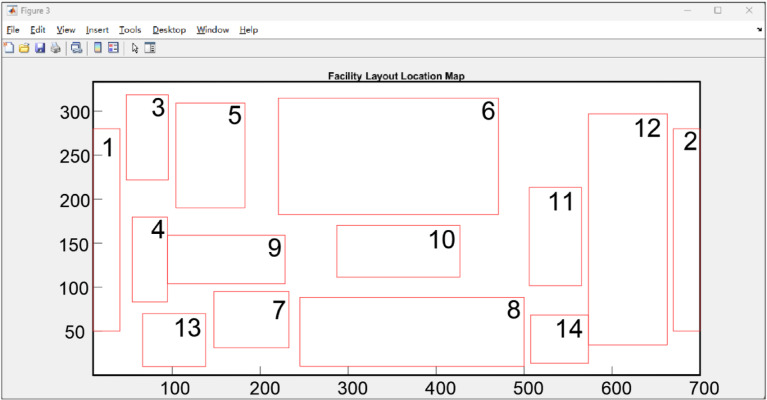
The result of algorithm outputs.

**Fig. 9:**
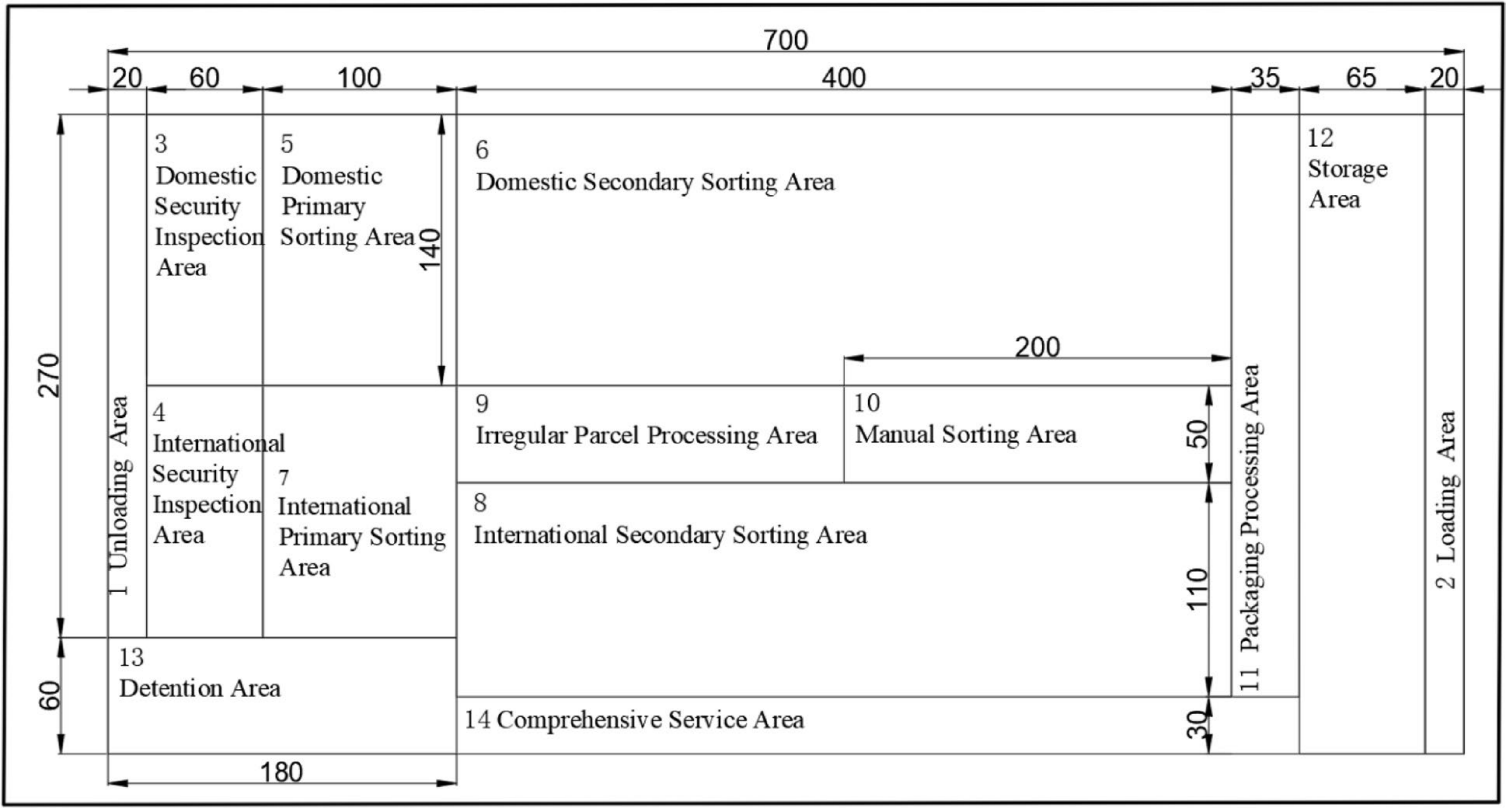
Aeronautical Logistics Center Layout Planning Diagram.

Based on the automatically generated layout results from the genetic algorithm, fine-tuning was performed on the outcome shown in Fig. 8 by considering practical factors such as space utilization efficiency and operational convenience to achieve a more rational solution. The final layout planning scheme is presented in Fig. 9.

**Fig. 10:**
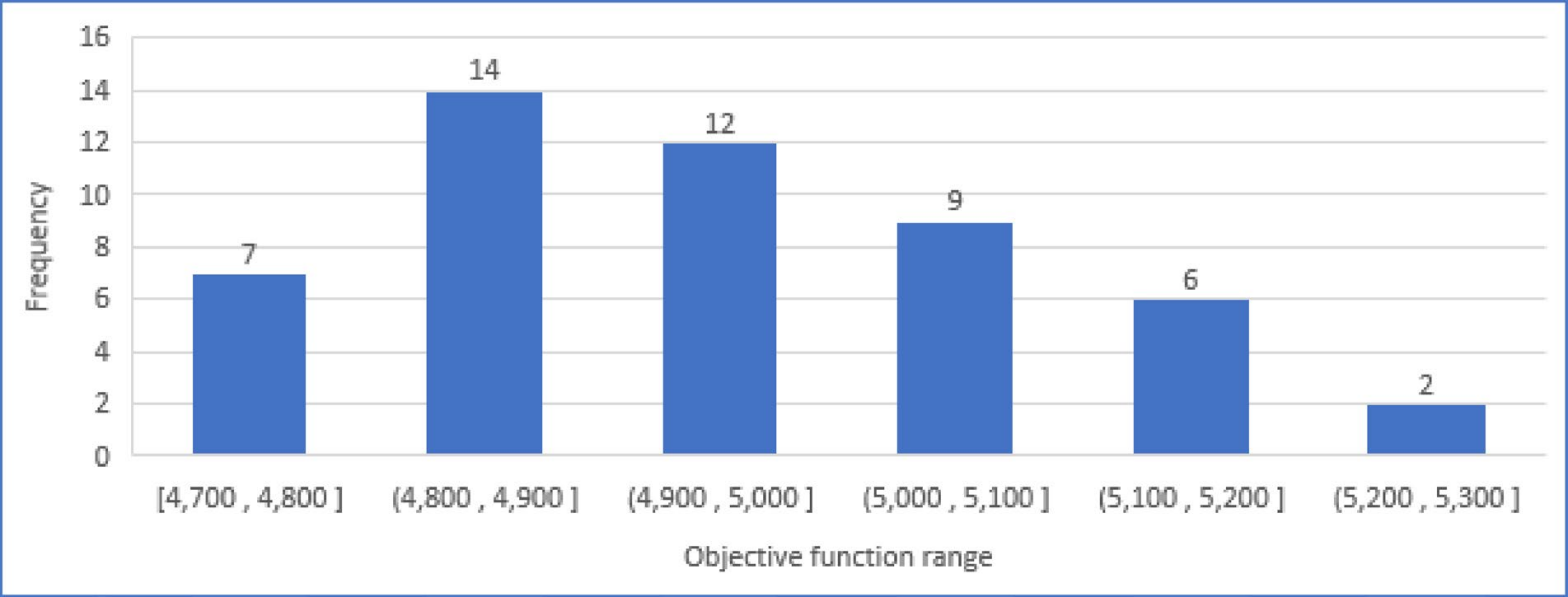
Histogram of Objective Function Values.

We conducted 50 independent runs for this instance under fixed input parameters. The statistical results of the optimal objective function values obtained from each run are shown in Fig. 10.

The statistical results show that the algorithm achieved a maximum value of 5463.95, a minimum value of 4885.63, a mean value of 5013.26, and a standard deviation of 156.323 over 50 independent runs. The small standard deviation indicates low variability in the results obtained by the genetic algorithm (GA), demonstrating high stability. Meanwhile, the mean value being close to the minimum value suggests that the algorithm is able to find near-optimal solutions in most cases, reflecting a high proportion of high-quality solutions. Additionally, 82% of the data points are concentrated within the range of [4800, 5200], further confirming the strong concentration and consistency of the results.

### Multi-scenario validation

**Fig. 11:**
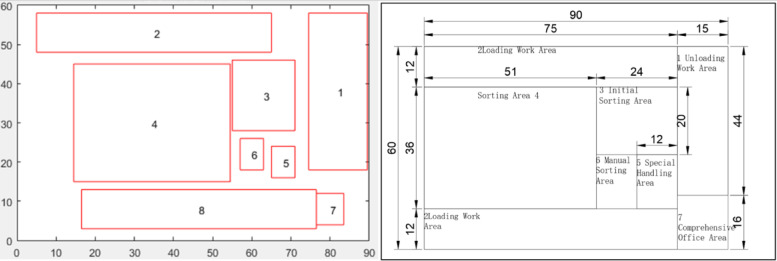
Small-scale air express sorting center.

Using a smaller-scale air express sorting center in another city as the experimental scenario, the planning area is considered as a rectangle measuring 90 units in length and 60 units in width. Based on the sorting process, the following functional areas are arranged: (1) unloading and receiving area, (2) loading operation area, (3) primary sorting area, (4) secondary sorting area, (5) special handling area, (6) manual sorting area, and (7) comprehensive office area. The minimum area of each functional zone is calculated according to logistics volume and sorting process, and the comprehensive interrelationships are determined. By applying the model and algorithm constructed in this study, the output shown in Fig. 11 is obtained, which verifies the validity of the model results.

## Discussion

This study aligns with facility layout planning theory and analyzes the demand characteristics of hub-level express parcel handling centers. By integrating key elements such as functional area dimensions and correlation values, this research developed a mathematical model based on equipment processing capacity and generated optimized layout solutions using the Systematic Layout Planning (SLP) method and a Genetic Algorithm. The results indicate that the proposed method can achieve intelligent layout planning for multi-level express handling centers. Compared to traditional manual layout methods, it can increase space utilization by 15–28% and significantly improve efficiency and accuracy.

The integration of Systematic Layout Planning (SLP) with the Genetic Algorithm ensures systematic optimization of functional areas, thereby enhancing resource allocation and operational performance. Furthermore, we conducted computational experiments on parcel sorting centers of various scales and types. The results showed low variability and high stability, indicating that the method consistently finds near-optimal, high-quality solutions in most cases. This demonstrates the validity and effectiveness of the proposed method, providing valuable decision support for the layout planning and upgrading of parcel handling centers.

## Conclusion

This study has limitations that require further exploration. First, the reliance on a single case study may restrict the generalizability of the findings. Future research should include multiple cases across diverse scenarios to validate the method’s robustness. Second, while the genetic algorithm is effective, its inherent constraints can yield multiple feasible solutions. Advanced optimization techniques or hybrid algorithms could be explored to refine the selection process and identify optimal layouts. Additionally, integrating simulation technologies would enable more detailed analysis of equipment requirements and operational flows, enhancing adaptability and precision. Moreover, as the amount of data increases, the initial solution construction by the current algorithm becomes progressively slower. Future work could consider other algorithms to improve the solution efficiency.

Despite these limitations, this study establishes an effective framework for parcel processing center layout planning, delivering 15–28% higher space optimization rates than traditional systematic layout planning alone, while improving efficiency and quality. The proposed method addresses challenges posed by increasing business volumes and time constraints through layout configurations that reduce wasted space by an average of 23%, offering a scalable solution for the evolving logistics industry. Future research should expand the application scope, incorporate real-time data, and explore emerging technologies such as artificial intelligence and digital twin technology to pursue space utilization improvements exceeding 40% while further optimizing logistics center layouts.

Furthermore, future research should explore extending the proposed integrated planning model to other logistics environments beyond express parcel hubs, such as cold chain logistics centers, e-commerce distribution warehouses, and urban last-mile delivery hubs. This would help validate the model’s versatility and adaptability across diverse logistics contexts and support broader applications in the evolving logistics industry.

This work was supported by National Key Research and Development Program of China (Grant No. 2022YFC3302200) and Beijing Social Science Foundation (Grant No. 22GLB018).

## Data Availability

The datasets analysed during the current study are not publicly available due to the project’s specific requirements for confidentiality, but are available from the corresponding author on reasonable request.Interested researchers may direct data requests to the author, Tao Wang, at jixiewangtao@163.com. Data requests will be evaluated based on reasonable academic access criteria.
